# APOE4 allele-specific associations between diet, multimodal biomarkers, and cognition among Puerto Rican adults in Massachusetts

**DOI:** 10.3389/fnagi.2023.1285333

**Published:** 2023-11-15

**Authors:** Yi Guan, Chia Hsin Cheng, Luis I. Bellomo, Sriman Narain, Sherman J. Bigornia, Mahdi O. Garelnabi, Tammy Scott, José M. Ordovás, Katherine L. Tucker, Rafeeque Bhadelia, Bang-Bon Koo

**Affiliations:** ^1^Department of Anatomy and Neurobiology, Boston University Chobanian and Avedisian School of Medicine, Boston, MA, United States; ^2^Department of Agriculture, Nutrition, and Food Systems, College of Life Sciences and Agriculture, University of New Hampshire, Durham, NH, United States; ^3^Department of Public Health, Zuckerberg College of Health Sciences, University of Massachusetts Lowell, Lowell, MA, United States; ^4^School of Medicine, Tufts University, Boston, MA, United States; ^5^Nutrition and Genomics Laboratory, J.M.-US Department of Agriculture Human Nutrition Research Center on Aging at Tufts University, Boston, MA, United States; ^6^IMDEA Alimentacion, Madrid, Spain; ^7^CIBER Fisiopatologia de la Obesidad y la Nutricion (CIBEROBN), Instituto de Salud Carlos III, Madrid, Spain; ^8^Department of Biomedical and Nutritional Sciences, Zuckerberg College of Health Sciences, University of Massachusetts Lowell, Lowell, MA, United States; ^9^Center for Population Health, University of Massachusetts Lowell, Lowell, MA, United States; ^10^Neuroradiology Section, Beth Israel Deaconess Medical Center, Harvard Medical School, Boston, MA, United States

**Keywords:** APOE, blood–brain barrier, neurite orientation dispersion and density imaging, diet, neuroinflammation, blood biomarker, aging, cognitive decline

## Abstract

**Background:**

Apolipoprotein E (APOE) is the strongest genetic risk factor for sporadic Alzheimer’s Disease (AD), and the ε4 allele (APOE4) may interact with lifestyle factors that relate to brain structural changes, underlying the increased risk of AD. However, the exact role of APOE4 in mediating interactions between the peripheral circulatory system and the central nervous system, and how it may link to brain and cognitive aging requires further elucidation. In this analysis, we investigated the association between APOE4 carrier status and multimodal biomarkers (diet, blood markers, clinical diagnosis, brain structure, and cognition) in the context of gene–environment interactions.

**Methods:**

Participants were older adults from a longitudinal observational study, the Boston Puerto Rican Health Study (BPRHS), who self-identified as of Puerto Rican descent. Demographics, APOE genotype, diet, blood, and clinical data were collected at baseline and at approximately 12th year, with the addition of multimodal brain magnetic resonance imaging (MRI) (T1-weighted and diffusion) and cognitive testing acquired at 12-year. Measures were compared between APOE4 carriers and non-carriers, and associations between multimodal variables were examined using correlation and multivariate network analyses within each group.

**Results:**

A total of 156 BPRHS participants (mean age at imaging = 68 years, 77% female, mean follow-up 12.7 years) with complete multimodal data were included in the current analysis. APOE4 carriers (*n* = 43) showed reduced medial temporal lobe (MTL) white matter (WM) microstructural integrity and lower mini-mental state examination (MMSE) score than non-carriers (*n* = 113). This pattern was consistent with an independent sample from the Alzheimer’s Disease Neuroimaging Initiative (ADNI) of *n* = 283 non-Hispanic White adults without dementia (mean age = 75, 40% female). Within BPRHS, carriers showed distinct connectivity patterns between multimodal biomarkers, characterized by stronger direct network connections between baseline diet/blood markers with 12-year blood/clinical measures, and between blood markers (especially lipids and cytokines) and WM. Cardiovascular burden (i.e., hypertension and diabetes status) was associated with WM integrity for both carriers and non-carriers.

**Conclusion:**

APOE4 carrier status affects interactions between dietary factors, multimodal blood biomarkers, and MTL WM integrity across ~12 years of follow-up, which may reflect increased peripheral-central systems crosstalk following blood–brain barrier breakdown in carriers.

## 1. Introduction

Apolipoprotein-E (APOE) is the strongest genetic risk factor for late-onset Alzheimer’s Disease (LOAD, age of onset after 65y), which accounts for more than 90% of all AD cases (Kunkle et al., 2019). In particular, the APOE ε4 allele (APOE4) has been shown to increase the risk of AD through multiple pathways, such as reduced β-amyloid clearance, increased microglial proinflammatory activation, disrupted glucose and lipid metabolism, synaptic dysfunction, and increased blood–brain barrier (BBB) permeability (Yamazaki et al., 2019; Serrano-Pozo et al., 2021). Moreover, APOE is critical for regulating lipid transport and metabolism, and may interact with dietary factors to alter AD risk (Yassine and Finch, 2020).

Brain white matter (WM) degeneration (reflecting oligodendrocyte malfunctioning and myelin loss) is a well-established early marker for normal brain aging or disease-related neurobiological changes and may be differentially affected by individual genetic (e.g., APOE4 carrier) status, medical conditions (e.g., cardiovascular burden), and lifestyle (e.g., diet) factors (Nasrabady et al., 2018; Habes et al., 2021). We previously showed, in a community-dwelling, longitudinal Puerto Rican older adult population (BPRHS: Boston Puerto Rican Health Study) that WM microstructural integrity (determined by diffusion-weighted magnetic resonance imaging) was especially susceptible to cardiovascular risk (CVR) factors such as hypertension and diabetes status (Guan et al., 2022). However, the role of APOE in this relationship remains unclear. A possible mechanism connecting perturbations to the peripheral system (i.e., dietary intervention) and brain structural changes in APOE4 carriers is through BBB breakdown. Recent *in vivo* human neuroimaging studies showed that APOE4 carriers had significantly higher BBB permeability, especially in the medial temporal lobe (MTL) region (i.e., hippocampus and parahippocampal gyrus), which corresponded to their cognitive status, and was independent of β-amyloid and tau burden (Nation et al., 2019; Montagne et al., 2020). Pericyte-deficient mice with prominent BBB leakage at 4-month-old also showed reduced WM integrity at 12-month-old (Montagne et al., 2018). Therefore, altered communications between the peripheral circulatory system and central nervous system (CNS) in APOE4 carriers may underlie their differential long-term response to lifestyle factors and contribute to immune and metabolic disruptions during midlife and AD pathogenesis.

In the current analysis, we examined associations between APOE4 carrier status, multimodal health factors (diet, blood biomarkers, CVR), MTL WM microstructure (as an early marker for brain aging related to BBB breakdown), and cognition in the longitudinal observational BPRHS cohort. The BPRHS enrolled self-identified Puerto Rican older adults without dementia living in the Greater Boston Area, who had clinical interviews and blood samples acquired at baseline and at the 12th year follow-up, with the addition of multimodal brain magnetic resonance imaging (MRI) at 12-year (Tucker et al., 2010; Guan et al., 2022). Measures were compared between APOE4 carriers and non-carriers using linear regression models, adjusting for covariates (i.e., age, sex, etc.). To examine possible ethnicity-specific effects related to APOE on brain WM and cognition, we additionally obtained APOE genotype, MRI, and cognitive data from an independent sample of non-Hispanic White participants from Alzheimer’s Disease Neuroimaging Initiative (ADNI) to perform the same group-level comparisons. Within the APOE4 carrier and non-carrier groups of BPRHS, we applied both the conventional univariate correlation analyses and a network-based approach to better characterize the complex interactions between multimodal variables. This multi-layer network model is inspired by the weighted network analysis that is particularly useful for examining relationships within high-dimensional datasets (Langfelder and Horvath, 2008; Horvath, 2011). Our work may facilitate future detailed investigation of distinct biological pathways connecting lifestyle factors and health outcomes in genetically diverse populations.

## 2. Materials and methods

### 2.1. Study cohort description

The BPRHS is a longitudinal study initiated in 2004 with the primary goal of studying the effect of psychosocial stress on various health outcomes (Tucker et al., 2010). The BPRHS (clinical trials registration number NCT01231958) was approved by the University of Massachusetts (UMASS) Lowell Institutional Review Board, IRB #17–143. The Tufts and Beth Israel Deaconess Medical Center (BIDMC) IRBs both ceded review to UMASS Lowell. Boston University (BU) determined that their roles were not considered human subject research. Written informed consent was obtained from all participants (or guardians of participants) in the study. All eligible participants self-identified as Puerto Rican, were able to answer questions in English or Spanish, were between 45 and 75 years of age, and lived in the Boston, MA, metropolitan region at the time of the study. Exclusion criteria included inability to answer questions due to serious health conditions, plans to move from the area, or Mini-Mental State Examination (MMSE) score ≤ 10. A total of 2,084 eligible participants were identified, from which 1,500 participants completed the baseline interviews. Participants had clinical interviews at baseline, and completed ~2.2, ~6.2, and ~12.7 years follow-ups. Multimodal MRI scans were obtained from a subset of participants who presented to the fourth follow-up (referred to as 12-year visit hereafter), performed on a 3-Tesla GE Signa HDx scanner (GE Healthcare, Milwaukee, WI) in BIDMC (Guan et al., 2022). The current analysis included participants with multimodal data, which include APOE genotype, non-imaging health-related information from different domains (dietary score, blood markers, clinical record, etc.) at the baseline visit, and brain imaging (T1-weighted MRI and diffusion MRI) and cognitive testing at the 12-year visit. A complete list of the non-imaging measures can be found in Table 1. Matching measures were obtained for non-Hispanic White participants from the ADNI cohort (Supplementary methods).

**Table 1:** List of non-imaging multimodal measures and their association with APOE4 carrier status.

**Time point**	**Variable domain**	**Variable name (Unit)**	**Network abbreviation**	**APOE4-** **(*n* = 113)**	**APOE4+** **(*n* = 43)**	**B** **(95% CI)**
**Baseline**	Diet	Healthy Eating Index 2005	HEI	72 (67–78)	72 (65–78)	0.72 (−2.6, 4.0)
		Healthy Eating Index 2005 excluding oil	HEI1	63 (57–69)	62 (57–68)	0.97 (−2.3, 4.2)
	Blood B Vitamins	Vitamin B6 (nm/L)	B6	48 (31–67)	46 (34–66)	−8.6 (−42, 25)
		Vitamin B12 (pg/mL)	B12	460 (343–663)	466 (364–665)	47 (−44, 138)
	Blood Fatty Acids	26 fatty acids in Supplementary Table S5	1–26	Refer to Supplementary Table S5		
**12-Year**	Blood Immune	C-reactive protein (mg/L)	CRP	2.5 (1.2–4.8)	3.0 (1.1–4.7)	−0.16 (−2.7, 2.4)
		Interleukin-6 (pg/mL)	IL-6	0.47 (0.07–1.0)	0.62 (0.19–1.0)	0.67 (−0.025, 1.4)
		TNF alpha (pg/mL)	TNFα	9.0 (7.2–11)	8.5 (6.3–11)	−0.85 (−3.8, 2.1)
	Blood Lipids	Total cholesterol (mg/dL)	Cholesterol	191 (156–222)	186 (152–204)	−9.0 (−24, 6.2)
		LDL Cholesterol (mg/dL)	LDL	108 (81–134)	100 (78–115)	−9.7 (−22, 2.3)
		HDL Cholesterol (mg/dL)	HDL	52 (43–61)	49 (40–61)	−2.0 (−7.1, 3.2)
		Triglycerides (mg/dL)	TG	131 (97–182)	139 (103–185)	7.3 (−25, 39)
	Other Blood Markers	Phosphorus (mg/dL)	Phosphorus	3.4 (3.1–3.9)	3.4 (3.1–4.0)	0.024 (−0.24, 0.29)
		Creatinine (mg/dL)	Creatinine	0.68 (0.57–0.87)	0.77 (0.54–0.91)	0.072 (−0.044, 0.19)
		Glucose (mg/dL)	Glucose	111 (98–134)	121 (100–141)	5.4 (−11, 22)
		Klotho (pg/mL)	Klotho	165 (17–1,079)	170 (10–3,065)	910 (−2,558, 4,379)
		Fibroblast growth factor 23 (nmol/L)	Fgf23	2.1 (1.1–4.8)	2.6 (1.8–4.7)	1.6* (0.25, 3.1)
	Clinical Measures	Hypertension and Diabetes status (0: none, 1: either, 2: both)	CVR	1 (0–2)	1 (1–2)	0.16 (−0.12, 0.43)
		BMI	BMI	31 (28–35)	32 (27–35)	−0.20 (−2.4, 2.0)

The above table listed the non-imaging measures (a total of 44 variables) acquired from BPRHS participants included in the current study. The domains and abbreviations were used for the network construction in Figure 3. Data for each group is presented as median (interquartile range). *Significantly different (corrected *p* < 0.05) between APOE4 carriers vs. non-carriers with linear regression model adjusting for age and sex (result presented as unstandardized regression coefficient B and 95% CI). *P*-values were corrected for multiple comparisons using permutation. APOE4– = non-carriers; APOE4+ = carrier.

### 2.2. Neuroimaging

#### 2.2.1. T1-weighted MRI

As described previously in Guan et al., 2022, the structural imaging data from BPRHS were obtained with General Electric (GE) 3 T MRI scanner for each participant with the following imaging parameters: repetition time (TR) = 7.6 ms, echo time (TE) = 3 ms, flip angle (FA) = 8, inversion time (TI) = 900 ms, slice thickness (ST) = 1.0 mm, total slice number = 164, field of view (FOV) = 25.6 cm, in-plane matrix (MX) = 256 × 256. From the above procedures, we extracted volumes of the left and right hippocampus, and total intracranial volume (ICV) (Fischl, 2012).

#### 2.2.2. Diffusion MRI

**Figure 1:**
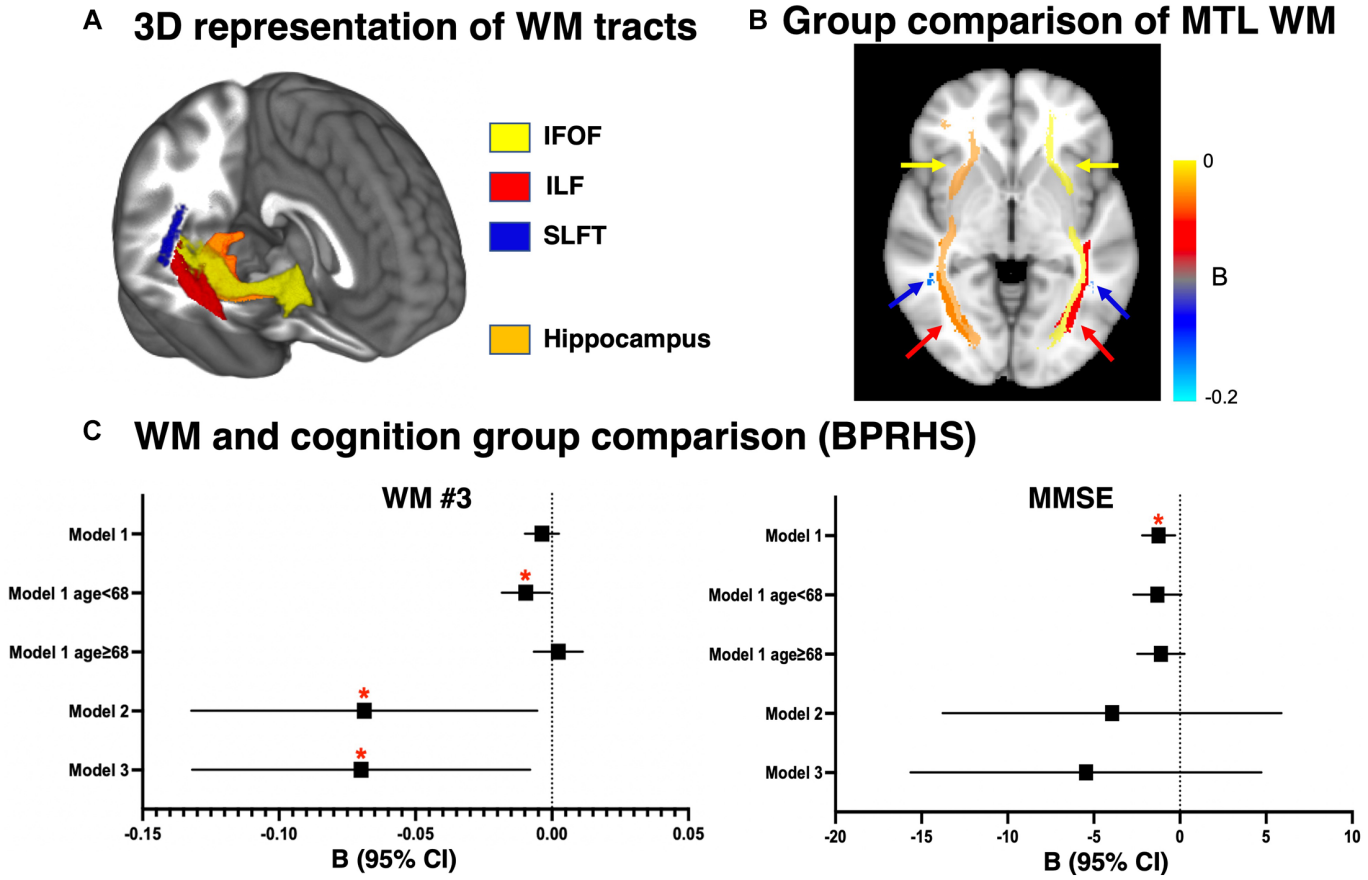
Anatomical view of medial temporal lobe white matter (WM) tracts and comparison between APOE4 carriers and non-carriers. **(A)** 3D brain rendering of the MTL WM tracts in the right hemisphere, in relation to the hippocampus. **(B)** Group-level statistical comparison of APOE4 carriers vs. non-carriers in the WM ND measure. Color scale corresponds to the regression coefficient B in model 2. The color of the arrows corresponds to the name of the tracts shown in **(A)**. **(C)** Group-level statistical comparison of APOE4 carriers vs. non-carriers in WM ND measure and MMSE score in the BPRHS cohort. The linear regression model included APOE4 carrier status as the main effect and additional covariates (Model 1: age and sex. Model 2: age, sex, and age × APOE4 interaction. Model 3: age, sex, age × APOE4, education and CVR). Additionally, education was included for all models with MMSE. *p < 0.05 after correction for multiple comparison using permutation method. BPRHS, Boston Puerto Rican Health Study; MTL, medial temporal lobe; ND, neurite density; WM, white matter; IFOF, inferior fronto-occipital fasciculus; ILF, inferior longitudinal fasciculus; SLFT, superior longitudinal fasciculus temporal portion.

Diffusion MRI data were obtained from participants based on the multishell sampling designed to provide shells of radius 1/3, 2/3, and 1 times the max *b*-values, 2,500 s/mm^2^, with 125 directional encodings (TR = 3,400 ms, TE = 73.4 ms, FA = 90, FOV = 24.0, MX = 256 × 256, ST = 2 mm). Microstructural diffusion measures were reconstructed from multi-shell diffusion MRI images containing 3 b-value encodings using Neurite Density Imaging (NDI), as previously described (Zhang et al., 2012; Cheng et al., 2020; Guan et al., 2022). From the NDI model, we extracted the neurite density index that measures the fraction of tissue composed of neurites which included axons/dendrites and tissue other than neurites. All NDI data were projected to a common space using the individual’s fractional anisotropy (FA) images for nonlinear registration and skeletonization. Next, mean NDI images were created and thinned to create a mean skeleton map that represents the centers of all tracts common to the group. Each subject’s aligned NDI data were then projected onto this skeleton and the resulting data was fed into voxel-wise cross-subject statistics. NDI measures including neurite density (ND) and orientation dispersion (OD) were extracted from 20 white matter regions of interest (ROIs). For the current analysis, we focused on the MTL tracts including left and right inferior fronto-occipital fasciculus (IFOF), inferior longitudinal fasciculus (ILF), and superior longitudinal fasciculus temporal portion (SLFT) (Figure 1A).

### 2.3. Multi-modal non-imaging measures

#### 2.3.1. Cognitive status

For BPRHS, cognitive function was assessed with a comprehensive battery of neuropsychological tests administered in the participant’s preferred language (98% in Spanish) by a trained research assistant (Fortuny et al., 1999). The current analysis included the mini-mental state examination (MMSE) score at the 12th year as a measure of general cognitive function and for comparison with the ADNI cohort.

#### 2.3.2. Diet, blood, and clinical markers in BPRHS

To study the relationship between diet, blood biomarkers and brain imaging outcomes, we obtained multimodal data from each BPRHS participant (Supplementary methods). In brief, dietary intake was measured with a validated food frequency questionnaire, and diet quality was determined using the Healthy Eating Index-2005 (HEI) (Tucker et al., 1998; Guenther et al., 2008). Blood samples were collected at each visit, as previously described, and were analyzed by ELISA using BioTek’s Epoch Microplate Reader (Gen5 Software empowered) or Clinical Chemistry analyzer (EasyRA), including C-reactive protein (CRP), tumor necrosis factor α (TNFα), interleukin-6 (IL-6), total cholesterol, LDL cholesterol (LDL), HDL cholesterol (HDL), triglycerides (TG), klotho, fibroblast growth factor 23 (FGF23), phosphorus, creatinine, glucose, vitamin B6, B12, and a total of 26 blood long chain fatty acids (Table 1; Supplementary Table S5; Tucker et al., 2010; Bigornia et al., 2016). Clinical measures included BMI and a CVR score (range 0–2) calculated by counting hypertension (systolic/diastolic blood pressure ≥ 140/90 mmHg or using antihypertensive medication) and diabetes status (fasting blood glucose ≥126 mg/dL or using medication) at the 12-year visit.

### 2.4. Statistical analysis

#### 2.4.1. Univariate group analysis

Demographic variables were compared between APOE4 carriers and non-carriers within BPRHS (or ADNI) using either t-test (for continuous variables such as age and BMI) or chi-square test (for categorical variables such as sex). Brain imaging measures (medial temporal lobe WM ND and OD, hippocampal volume) and MMSE were compared between APOE4 carriers and non-carriers within BPRHS (or ADNI) using general linear regression models (Models 1–3), adjusting for differing sets of covariates. In addition to the APOE4 main effect, model 1 adjusted for age and sex, model 2 adjusted for age, sex, and age × APOE4 interaction, and model 3 adjusted for age, sex, age×APOE4 interaction, education, and CVR score. We also included education level for all models comparing MMSE, and intracranial volume (ICV) was included for comparisons of hippocampal volume. For comparing hippocampal volume between ADNI APOE4 carriers vs. APOE4 non-carriers, we additionally included MRI magnetic strength (1.5-Tesla or 3-Tesla) as a covariate. Results were presented as unstandardized regression coefficients (B), 95% confidence interval (CI), and *p*-value (after adjusting for the effect of multiple comparison using permutation-based method) (Groppe et al., 2011). Within the BPRHS, we also compared non-imaging markers (i.e., HEI, blood cytokines, etc.) between APOE4 carriers and non-carriers, using linear regression models adjusted for age and sex. Within APOE4 carrier or non-carrier group, we examined associations between all baseline and 12-year multimodal variables using partial correlation, adjusting for age and sex. Correlations were presented as correlation coefficient R and adjusted *p*-value (after permutation correction). All analyses were conducted using Matlab software, version 2022b (MathWorks, Inc).

#### 2.4.2. Multivariate network analysis

Multivariate network analysis was used to study interactions between multimodal biomarkers, which can help to understand the effect of peripheral perturbation (i.e., diet) on the CNS during aging. To understand a complex system underlying cognitive aging in the BPRHS, we applied a multi-layer network analysis model based on the well-established weighted correlation network analysis concept (details in Supplementary Methods; Zhang and Horvath, 2005; Horvath, 2011). In brief, a lower-level network (LLN) based on correlation matrix (dimension 50 × 50, including 44 non-imaging variables and 6 imaging variables) was constructed to represent connectivity between each pair of variables. Soft thresholding (power function and Topological Overlap Measure) was applied to the matrix to emphasize strong connections and remove spurious connections. Each connection in the LLN has a weight (W), indicating the connectivity strength. To improve visualization of extensive network structure from the high dimensional data, we used biologically predefined domains (i.e., blood lipid, cytokines, etc.) and hierarchical cluster analysis to define modules of highly interconnected variables. The resulting upper-level network (ULN) represented average connectivity (W^ave^) between modules. To quantitatively compare W^ave^ of matching edges between carriers and non-carriers and account for bias related to imbalanced group sizes, we applied a resampling strategy to randomly subsample 34 individuals from each group (equivalent to 80% of APOE4 carriers) with 1,000 iterations and compared using the Mann–Whitney U test and Mood’s median test. Network analyses were conducted using an in-house pipeline written in R (version 4.2.3).

## 3. Results

### 3.1. Participant characteristics

Out of the 1,500 BPRHS participants, 200 individuals have completed brain MRI by the time of the current study, where 44 were excluded due to missing genetic or blood marker data. The remaining 156 BPRHS participants (mean age 68 years, 77% female) were included in the current analysis, which consisted of 113 APOE4 non-carriers (mean age 68 years, 78% female) and 43 APOE4 carriers (mean age 68 years, 74% female, 21% homozygous) (Table 2), with a mean follow-up period of 12.7 years. There were no reports of dementia or other neurological diseases in any participants at baseline or at the time of MRI (12-year). Within this cohort, there were no significant differences regarding age, sex ratio, education level, proportion of hypertension, diabetes, or BMI between APOE4 carriers and non-carriers at the time of MRI.

**Table 2:** Participant characteristics.

	BPRHS		
Variable	APOE4 non-carrier (*n* = 113)	APOE4 carrier (*n* = 43)	All (*n* = 156)
Age, mean ± SD, y	68 ± 6.7	68 ± 6.8	68 ± 6.7
Hispanic, *n* (%)	113 (100)	43 (100)	156 (100)
Female, *n* (%)	88 (78)	32 (74)	120 (77)
Education ≥ 9th grade, *n* (%)	64 (54)	27 (64)	91 (59)
Hypertension, *n* (%)	74 (67)	30 (75)	104 (69)
Diabetes, *n* (%)	38 (35)	17 (41)	55 (36)
BMI, mean ± SD	32 ± 5.9	32 ± 6.6	32 ± 6.1

Data are presented as count (%) or mean ± standard deviation. BMI, body mass index; BPRHS, Boston Puerto Rican Health Study; SD, standard deviation.

From ADNI, we included 283 non-Hispanic White participants without dementia: 160 APOE4 non-carriers (mean age 75 years, 43% female) and 123 APOE4 carriers (mean age 75 years, 36% female, 20% homozygous) (Supplementary Table S1). Comparing all BPRHS and ADNI individuals, the BPRHS sample had fewer carriers (*p* = 0.001), younger age (*p* < 0.0005), more females (*p* < 0.0001), fewer individuals with ≥9th-grade education (*p* < 0.0001), higher prevalence of hypertension (*p* < 0.0001) and diabetes (*p* < 0.0001), and higher BMI (*p* < 0.0005).

### 3.2. Multimodal biomarker comparisons in APOE4 carriers vs. non-carriers

For the BPRHS, MTL WM tract (bilateral IFOF, ILF, SLFT, Figure 1A) microstructural integrity was measured using diffusion MRI measures (ND, OD) and was compared between APOE4 carriers and non-carriers (Table 3). Model 1 (adjusted for age and sex) did not reveal significant differences between APOE4 carriers and non-carriers in ND or OD. Stratifying the sample into relatively younger (*n* = 78) and older (*n* = 78) individuals, based on mean age (68 years) showed lower WM ND in bilateral ILF (Left: *p* = 0.03, Right: *p* = 0.04) and bilateral SLFT (Left: *p* = 0.03, Right: *p* = 0.01) in carriers <68 years. Model 2 additionally accounted for interaction between age×APOE4 and showed lower ND in carriers in bilateral ILF (Left: *p* = 0.03, Right: *p* < 0.05) and right SLFT (*p* = 0.04) (Figure 1B), as well as higher OD in right ILF and left SLFT (Table 3) (Operto et al., 2019; Sun et al., 2020). In model 3, the same tracts remained clearly different after adjusting education and CVR (Figure 1C). Hippocampal volume measures did not show differences in any models. MMSE was lower in APOE4 carriers only in model 1 (*p* = 0.01), but not after additional covariates were included (Figure 1C). Based on the similar key ROIs identified in ND and OD, our subsequent correlation and network analyses of WM were conducted using ND. Comparing non-imaging biomarkers (i.e., HEI, blood cytokines, lipids, etc.) between APOE4 carriers and non-carriers, group differences were found for blood FGF23 concentration (*p* = 0.02) and arachidic acid (*p* = 0.03) (Table 1; Supplementary Table S5).

**Table 3:** The association of APOE4 carrier status with brain white matter tracts, MMSE, and hippocampal volume in BPRHS.

	**BPRHS (*n* = 156)**				
**Variable**	**Model 1** **All**	**Model 1**	**Model 1**	**Model 2** **All**	**Model 3** **All**
**B (95% CI), *P***		Age < 68 (*n* = 78)	Age ≥ 68 (*n* = 78)		
**WM ND**					
Left IFOF	−0.004 (−0.011, 0.003)	−0.008 (−0.017, 0.002)	0.000 (−0.009, 0.010)	−0.035 (−0.10, 0.033)	−0.030 (−0.10, 0.038)
	0.26	0.11	0.97	0.31	0.38
Right IFOF	−0.003 (−0.009, 0.004)	−0.006 (−0.014, 0.003)	0.001 (−0.009, 0.010)	−0.026 (−0.089, 0.038)	−0.025 (−0.088, 0.038)
	0.42	0.19	0.88	0.42	0.43
Left ILF	−0.004 (−0.010, 0.003)	**−0.010 (−0.018, −0.001)**	0.002 (−0.007, 0.011)	**−0.069 (−0.13, −0.005)**	**−0.070 (−0.13, −0.008)**
	0.25	**0.032**	0.61	**0.034**	**0.027**
Right ILF	−0.004 (−0.010, 0.003)	**−0.010 (−0.019, 0.000)**	0.002 (−0.007, 0.011)	**−0.067 (−0.010, 0.003)**	**−0.068 (−0.143–0.002)**
	0.25	**0.044**	0.60	**0.048**	**0.044**
Left SLFT	−0.010 (−0.021, 0.000)	**−0.018 (−0.033, −0.002)**	−0.004 (−0.019, 0.011)	−0.076 (−0.19, 0.034)	−0.077 (−0.19, 0.036)
	0.056	**0.028**	0.60	0.17	0.18
Right SLFT	−0.010 (−0.022, 0.002)	**−0.023 (−0.040, −0.006)**	0.003 (−0.014, 0.020)	**−0.13 (−0.25, −0.007)**	**−0.14 (−0.26, −0.013)**
	0.11	**0.009**	0.72	**0.038**	**0.031**
**WM OD**					
Left IFOF	0.002 (−0.001, 0.004)	0.001 (−0.003, 0.004)	0.002 (−0.001, 0.005)	0.004 (−0.024, 0.025)	−0.001 (−0.027, 0.024)
	0.19	0.63	0.21	0.97	0.91
Right IFOF	0.000 (−0.004, 0.005)	0.002 (−0.004, 0.009)	−0.002 (−0.008, 0.003)	0.044 (−0.001, 0.089)	0.044 (−0.002, 0.090)
	0.031	0.49	0.41	0.054	0.061
Left ILF	−0.002 (−0.007, 0.004)	0.001 (−0.003, 0.006)	−0.006 (−0.016, 0.005)	0.026 (−0.030, 0.083)	0.030 (−0.028, 0.088)
	0.54	0.52	0.28	0.36	0.31
Right ILF	0.002 (−0.003, 0.007)	0.008 (−0.001, 0.016)	**−0.006 (−0.010, 0.001)**	**0.083 (0.033, 0.132)**	**0.088 (0.036, 0.14)**
	0.53	0.068	**0.03**	**0.001**	**0.001**
Left SLFT	0.005 (−0.001, 0.010)	0.009 (−0.002, 0.020)	−0.001 (−0.002, 0.001)	**0.066 (0.009, 0.122)**	**0.066 (0.005, 0.126)**
	0.11	0.099	0.29	**0.024**	**0.033**
Right SLFT	0.000 (−0.001, 0.001)	0.000 (−0.001, 0.002)	−0.000 (−0.001, 0.000)	0.004 (−0.004, 0.011)	0.004 (−0.004, 0.011)
	0.84	0.48	0.38	0.32	0.33
**GM volume**					
Left hippocampus	15 (−113, 144)	62 (−127, 250)	−48 (−226, 130)	609 (−722, 1941)	336 (−999, 1,670)
	0.81	0.52	0.59	0.37	0.62
Right hippocampus	14.5 (−126, 155)	48 (−145, 242)	−24 (−231, 183)	−175 (−1,631, 1,281)	−374 (−1837, 1,088)
	0.84	0.62	0.82	0.81	0.61
Average hippocampus	15 (−114, 144)	55 (−131, 241)	−36 (−220, 148)	217 (−1,126, 1,561)	−19 (−1,363, 1,324)
	0.82	0.56	0.70	0.75	0.98
**Cognitive test**					
MMSE	**−1.2 (−2.2, −0.28)**	−1.3 (−2.7, 0.084)	−1.1 (−2.5, 0.27)	−3.9 (−14, 5.9)	−5.5 (−16, 4.7)
	**0.012**	0.065	0.11	0.43	0.29

Statistical test results are presented for APOE4 carriers vs. non-carriers with unstandardized regression coefficient B (95% confidence interval). *P*-values were corrected for multiple comparisons using the permutation-based method. While studying the APOE4 main effect, we adjusted for different sets of covariates in 3 models: model 1 (age and sex), model 2 (age, sex, and age × APOE4 interaction), and model 3 (age, sex, age × APOE4, education, and CVR). Additionally, education was included for all models with MMSE, and intracranial volume (ICV) was included for those with hippocampal volume. BPRHS, Boston Puerto Rican Health Study; ND, neurite density; OD, orientation dispersion; WM, white matter; GM, gray matter; IFOF, inferior fronto-occipital fasciculus; ILF, inferior longitudinal fasciculus; SLFT, superior longitudinal fasciculus temporal portion; MMSE, mini-mental state examination. Bold values are for measures with *p* < 0.05.

For ADNI participants, due to the lack of similar multi-B diffusion sequence, we estimated WM integrity using CSF p-Tau and p-Tau/t-Tau ratio, as suggested by previous papers (Supplementary Figure S1; Strain et al., 2018; Raghavan et al., 2022). Both the inverses of p-Tau and p-Tau/t-Tau were lower in carriers than non-carriers in all models, suggesting similar group patterns as seen in the BPRHS WM measures. Hippocampal volumes and MMSE were lower in APOE4 carriers in model 1 but were attenuated with additional covariates included in models 2 and 3 (Supplementary Figure S1; Supplementary Table S2).

### 3.3. Associations between diet, blood markers and brain WM integrity in APOE4 carriers and non-carriers

**Figure 2:**
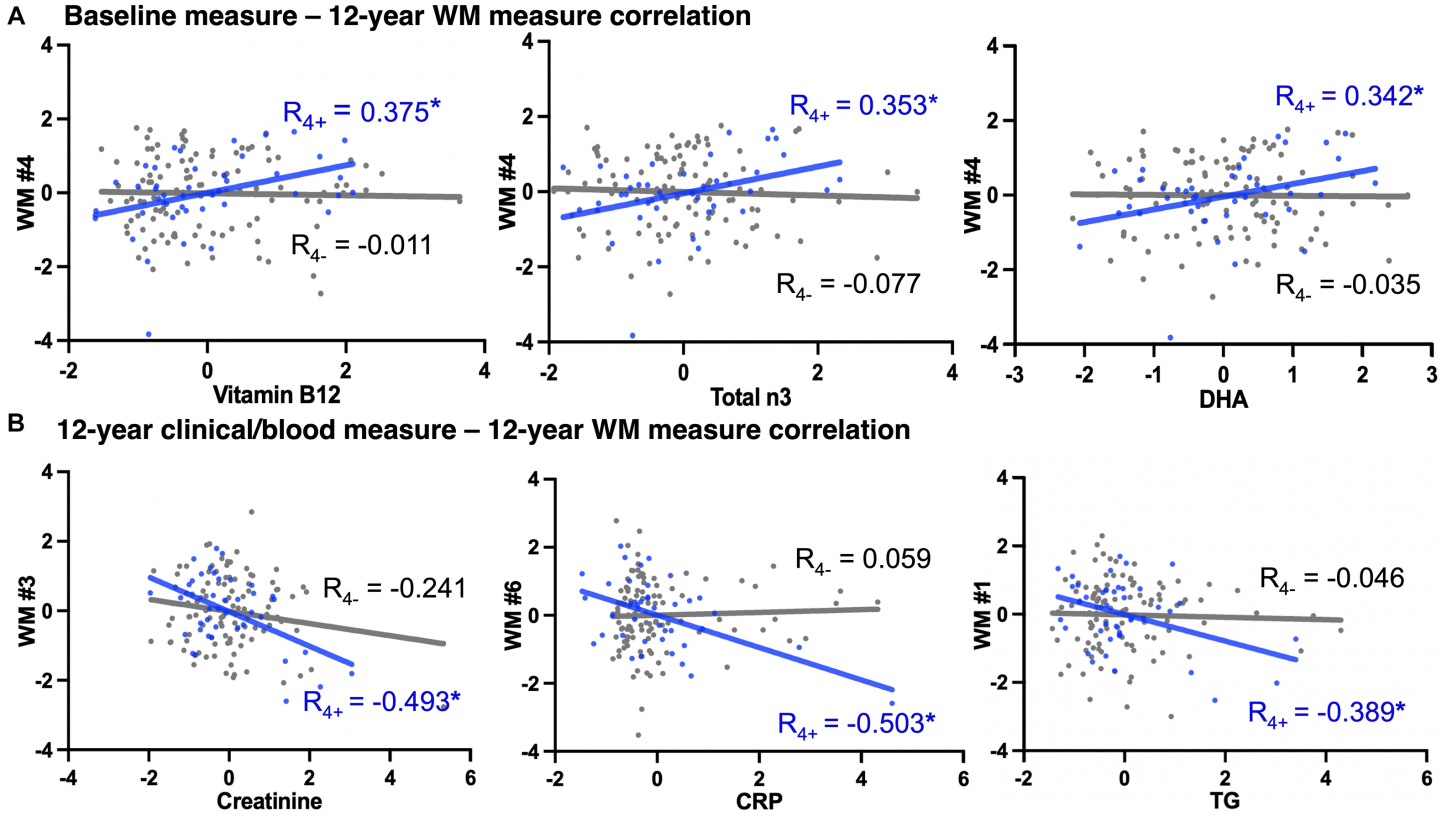
APOE4 carrier status-specific associations between non-imaging measures to 12-year WM measures. A selection of correlations (adjusted for age and sex, *indicate *p* < 0.05) with largest coefficient R among all significant pair-wise correlations. The *x*- and *y*-axes were the residual deviance of each variable after adjusted for age and sex. Blue dots/line = APOE4 carriers, gray dots/line = APOE4 non-carriers. **(A)** Associations between baseline non-imaging (i.e., blood) measures and 12-year brain WM measures. **(B)** Associations between 12-year non-imaging measures and 12-year brain WM measures. WM, white matter; WM #1/2, left/right inferior fronto-occipital fasciculus; WM #3/4, left/right inferior longitudinal fasciculus; WM #5/6, left/right superior longitudinal fasciculus temporal portion; DHA, docosahexaenoic acid; CRP, C-reactive protein; TG, triglyceride. Complete lists of variable abbreviations can be found in Table 1 and Supplementary Table S5.

Relationships between multimodal variables were examined using pair-wise partial correlation analyses between (1) baseline diet/blood marker and 12-year brain WM ND, (2) baseline diet/blood marker and 12-year clinical/blood measures, and (3) 12-year clinical/blood measures and 12-year WM ND, in the carrier and non-carrier groups. Our results showed more significant associations between baseline non-imaging and brain WM measures in APOE4 carriers (Supplementary Tables S3, S4). We found associations between blood vitamin B12 and right ILF, blood total n3 fatty acids and right ILF, and blood G-Linolenic acid and left IFOF among APOE4 carriers (*p* < 0.05), but not in non-carriers (Figure 2A). Similarly, we observed correlations between 12-year clinical/blood measures and 12-year WM with larger magnitudes of *R* value in APOE4 carriers than non-carriers (Figure 2B).

**Figure 3:**
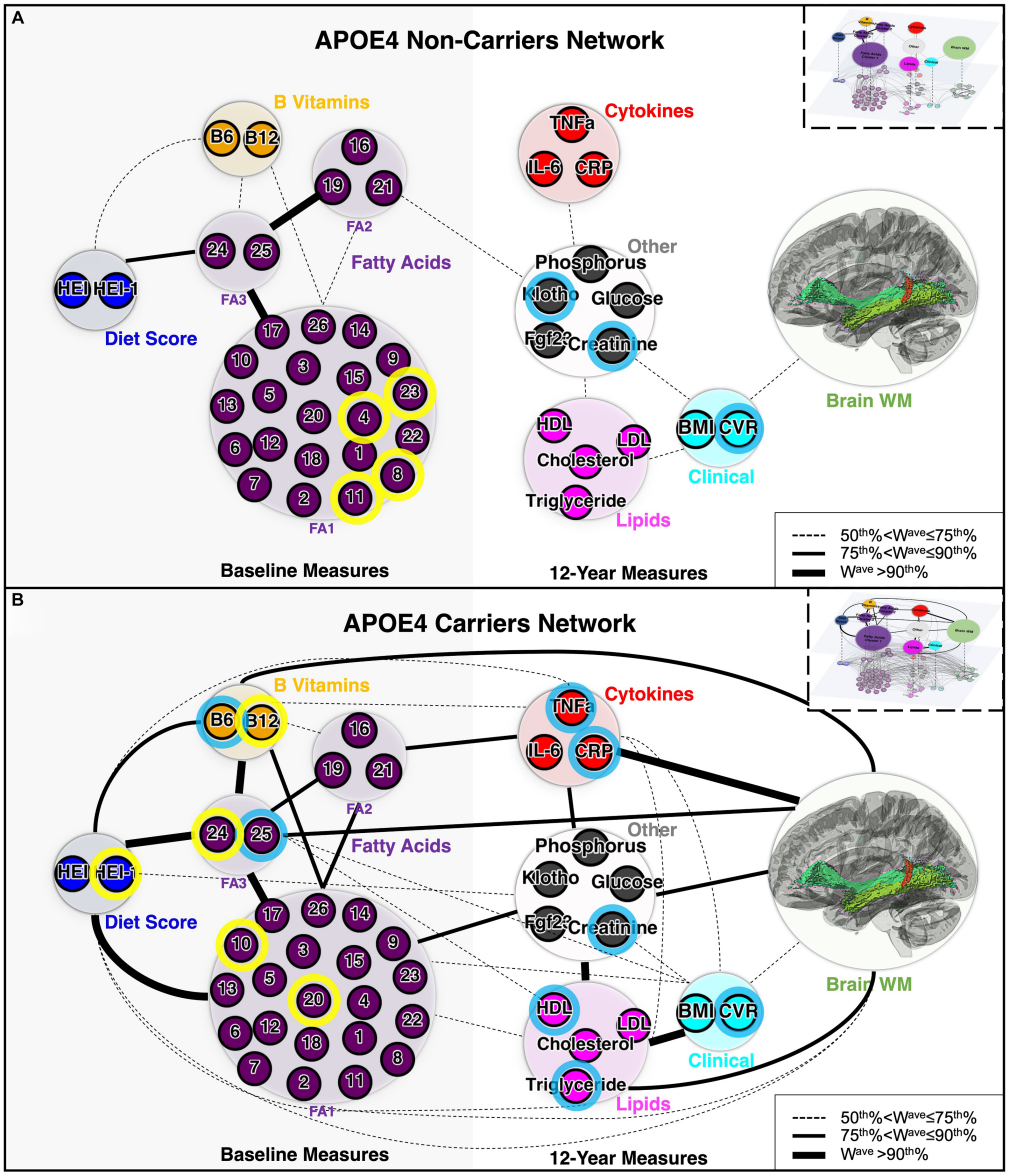
Architecture of APOE4 carrier and non-carrier networks. **(A)** Network for APOE4 non-carriers and **(B)** network for APOE4 carriers. The small circles represent individual variables (nodes of the lower-level network). Nodes with blue outlines were significantly correlated with WM based on univariate correlation analysis. Nodes with yellow outlines were significantly correlated with both WM and 12-year blood/clinical measures, based on univariate correlation analysis. The large circles represent each module (domains or cluster) of variables (nodes of the upper-level network, Q). The colors of the circles represent the biological domain of the multimodal variables. The thickness of the lines represents the weight (connectivity strength), W^ave^, of the ULN connection (edge) between a pair of nodes, with cut-off points defined based on its percentile rank among all connections. Boxes in the upper right corner provide a schematic of the LLN (connections between individual variables) and the ULN (connections between each domain/cluster of variables). LLN, lower-level network; ULN, upper-level network; WM, white matter; FA1, fatty acid cluster 1; FA2, fatty acid cluster 2; FA3, fatty acid cluster 3. The complete list of abbreviations used can be found in Table 1 and Supplementary Table S5.

**Figure 4:**
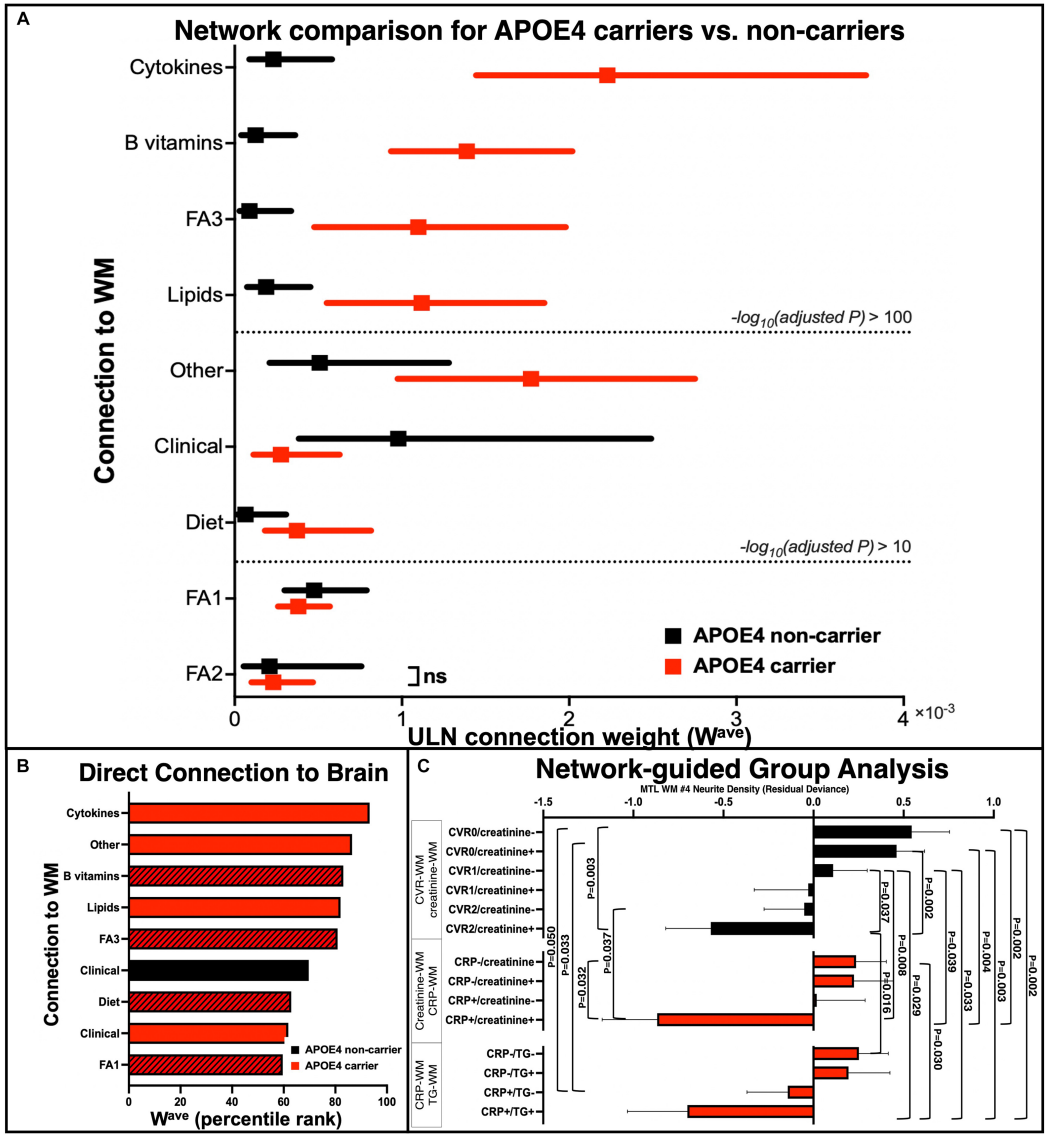
Quantitative group comparisons based on the APOE4 carrier and non-carrier networks. **(A)** Comparison of APOE4 carriers’ and non-carriers’ ULN WM-related connections (W^ave^). After iterative random subsampling (*n* = 34 from each group selected per iteration, 1,000 iterations, data presented as median and interquartile range), group comparison of W^ave^ was conducted using Mood’s median test (and Mann–Whitney *U* test as an alternative). Dashed line represents cut-off points based on FDR-adjusted *p*-value (ns = not significant based on adjusted *p* < 0.05). **(B)** Direct connections between non-imaging measures to brain WM measure of both carrier and non-carrier networks, ranked from top to bottom according to highest to lowest W^ave^. Black bar: APOE4 non-carrier network; Red bar: APOE4 carrier network; Shaded bar: baseline measures. **(C)** Follow-up group analysis based on specific network pathways. We selected pairs of key nodes for subgroup definitions and performed group comparisons on their WM ND of right ILF (the *x*-axis is the residual deviance of ND after adjusting for age and sex). Black color: APOE4 non-carrier; red color: APOE4 carrier. *p*-values were corrected for multiple comparisons using permutation. ULN, upper-level network; WM, white matter; FA1, fatty acid cluster 1; FA2, fatty acid cluster 2; FA3, fatty acid cluster 3; MTL, medial temporal lobe; WM #4, right inferior longitudinal fasciculus.

To analyze association patterns more efficiently in high dimensional datasets with multimodal variables, we constructed multi-layer connectivity networks specific to each APOE group, based on the correlation strengths (Horvath, 2011). The resulting networks showed distinct connectivity patterns between APOE4 carriers and non-carriers (Figure 3), with three similarities between APOE4 carrier and non-carrier networks. First, from the hierarchical cluster analysis of 26 fatty acids, three clusters with consistent components were identified in both APOE4 carrier and non-carrier groups (Supplementary Figure S2). While 21 of the 26 fatty acids were clustered together (FA1), a distinct cluster consisting of behenic acid, lignoceric acid and nervonic acid was identified as FA2, and DHA and total n3 as FA3. Second, direct connections between 12-year clinical (BMI and CVR) and WM nodes were observed for both carriers and non-carriers. Third, both groups had overlapping direct connections between baseline measure nodes (i.e., diet – FA3, diet – B vitamins, three FA clusters, etc.), and between 12-year non-imaging measure (i.e., cytokines – other, lipids – clinical, etc.). In contrast, carrier and non-carrier networks differed, as there were multiple direct pathways between non-imaging and WM in carriers, whereas the only direct connection for non-carriers was clinical - WM. Overall, there were more strong direct connections between non-imaging modules in the carrier network (Figure 4B). There were also more direct pathways between non-imaging nodes in APOE4 carriers, such as diet score – FA1, and B vitamins – FA2. Overall, for the non-carrier network, clinical measures (BMI and CVR) were the only node directly linked to WM, mediating the indirect link for several other non-imaging nodes with WM, whereas for carriers, clinical measures and non-imaging nodes were directly associated with WM. These results suggest potentially enhanced interactions between the peripheral circulatory system and the CNS in APOE4 carriers.

We next sought to quantitatively compare the ULN connection strength (W^ave^) of the nine types of WM connections between APOE4 carriers and non-carriers (using iterative random subsampling and Mood’s median test/Mann–Whitney *U* test). We found that W^ave^ was different between carriers and non-carriers in all but the FA2 – WM connection (Figure 4A). Specifically, the W^ave^ of cytokine – WM showed the most significant increase in APOE4 carriers than non-carriers. The Mann–Whitney U and Mood’s median tests consistently highlighted the same features.

We additionally applied group-level statistics by selecting key nodes/edges to explore specific network structures. Group stratifications based on the combination of APOE4 status and biomarkers accurately described different levels of WM alterations (Figure 4C). For instance, non-carriers with both hypertension and diabetes (CVR = 2) and higher blood creatinine (creatinine+) had significantly reduced WM integrity, relative to non-carriers who had CVR = 1 (hypertension or diabetes) and normal blood creatinine (creatinine-), while non-carriers who had CVR = 0 and creatinine- showed the highest WM integrity. For carriers, groups based on blood CRP and creatinine/triglycerides showed a similar pattern: those who were abnormally high on both factors showed the lowest WM integrity, whereas those who were normal on both factors showed the highest WM integrity. This suggests that APOE4 carrier status could not fully explain the heterogeneity of brain WM integrity and that it might be improved by considering additional factors, such as blood biomarkers.

## 4. Discussion

The etiology of LOAD extends beyond simple monogenic origin and likely involves multiple susceptibility genes and complex gene–environment interactions, where stochastic factors (e.g., lifestyle, pollutants) play increasingly relevant roles in the pathogenesis (Frisoni et al., 2022). Previous studies suggest that APOE4 carrier status is associated with a higher risk of AD through both amyloid- and tau-dependent and independent pathways, such as increasing neuroinflammation, disrupting glucose and lipid metabolism, and reducing neurovascular integrity (Yamazaki et al., 2019). Moreover, the APOE genotype may interact with diet to further impact the risk of AD (Yassine and Finch, 2020). However, the precise relationship between APOE4 carrier status and the brain, and how it may interact with dietary factors and peripheral blood markers to contribute to AD-related neuropathology remain unclear. In a sample of Puerto Rican older adults without dementia, followed longitudinally for more than 10 years, we studied the association between baseline dietary factors, blood biomarkers, 12th year brain imaging markers and cognitive status in APOE4 carriers and non-carriers. Our results showed that: (1) The APOE4 allele is associated with reduced MTL WM microstructural integrity and cognitive performance, (2) cardiovascular burden is an important factor for brain WM microstructural integrity in both APOE4 carriers and non-carriers, (3) in APOE4 carriers only, we observed extensive connections between multimodal biomarkers across timepoints, suggesting increased peripheral circulatory system and CNS interactions that may be reflective of BBB breakdown. Our results corroborate previous evidence and suggest that carriers may respond differently to dietary intervention, and immune and metabolic disruptions during midlife, compared to non-carriers, contributing to brain structural changes and cognitive decline during aging.

Brain WM degeneration, underlying myelin damage and oligodendrocyte malfunctioning are sensitive and early markers of normal and pathological brain aging (Cox et al., 2016; Nasrabady et al., 2018). Previous studies in the non-human primate model of aging also showed prominent WM degeneration in the dorsal prefrontal cortex, area 46, and anterior cingulate cortex without any significant gray matter (GM) atrophy (Luebke et al., 2010; Peters and Kemper, 2012). By comparing WM and cognitive measures between APOE4 carriers and non-carriers without dementia, we observed significantly lower MTL WM tract integrity in carriers from the BPRHS, even after accounting for the effect of CVR (Figure 1B). A similar pattern was seen in ADNI individuals without dementia, using the alternative marker for MTL WM, p-Tau (Supplementary Figure S1). APOE4 carriers also performed significantly worse on MMSE than non-carriers. Our finding is consistent with earlier diffusion tensor imaging (DTI) studies showing that APOE4 carriers had lower fractional anisotropy and higher mean diffusivity (indicating loss of WM microstructural integrity) in the medial temporal WM than non-carriers, and with greater WM hyperintensity volumes regardless of AD diagnosis (Sudre et al., 2017). Brain tissue analyses of humans and rodents carrying APOE4 also showed prominent oligodendrocyte injuries and myelin loss (Blanchard et al., 2022). We also observed a significant age×APOE4 interaction effect, suggesting a non-linear effect of APOE on WM changes during aging (Operto et al., 2019; Sun et al., 2020). Our findings support WM degeneration as a sensitive indicator for detecting early and subtle impacts of APOE4 on brain aging.

On the other hand, we observed a significant APOE4 effect on hippocampal volume among ADNI participants, but not in BPRHS (Table 2 and Supplementary Table S2). Indeed, findings on APOE4 and GM atrophy have been inconsistent, which may be due to heterogeneous study populations. APOE4 carrier status was associated with lower hippocampal and MTL volumes among older adults with mild cognitive impairment (MCI) or AD, but not among young or middle-aged adults without dementia (Filippini et al., 2009; Hostage et al., 2014; Emrani et al., 2020). Longitudinal studies have shown that APOE4 is associated with accelerated hippocampal atrophy during later aging (marginal significance starting at age of 57 years) (Mishra et al., 2018). These findings suggest that APOE4-related GM changes may only be reliably detected by *in vivo* neuroimaging at an older age, or when prominent AD symptoms have already manifested. However, the focus of APOE studies on older and already impaired individuals may overlook how it modulates the early stages of AD, when treatments can be more effective.

The gene–environment interaction (between the APOE4 allele and lifestyle factors) may explain the different trajectories of WM degeneration and cognitive decline during aging. We found significant associations between diet, peripheral blood, B vitamins, lipid and cytokine concentrations, and brain WM integrity in APOE4 carriers (Figures 2, 3). Only among carriers was HEI at baseline positively correlated with WM integrity at 12 years, consistent with previous reports of carriers responding differently to dietary interventions and peripheral perturbations (i.e., weight loss) on cognitive function (Yassine and Finch, 2020). We also observed stronger associations between blood biomarkers (i.e., blood cytokines and lipids) and WM in APOE4 carriers, including between CRP and WM, between triglycerides and WM, etc. (Figure 2), while a direct link between CVR and WM integrity was found in both APOE groups (Figure 3), consistent with our previous analysis (Guan et al., 2022). Recent transcriptomic studies of human iPSCs suggest that APOE4 may disrupt lipid metabolism by glial cells, and APOE4-related demyelination was mitigated by cholesterol lowering treatment (Blanchard et al., 2022; Tcw et al., 2022). Indeed, lifestyle factors, such as a high-fat/high-sugar diet, are associated with higher inflammatory cytokines and cholesterol, contributing to metabolic and cardiovascular disorders and elevated risk of LOAD (Oblak et al., 2022). A recent large-scale study confirmed increased odds of AD, hypercholesterolemia, and ischemic heart disease in APOE4 carriers than in non-carriers (Lumsden et al., 2020). Interestingly, we found blood total n3 and DHA levels to positively correlate with MTL WM integrity in APOE4 carriers, consistent with previous evidence suggesting their critical role in brain structure and function (Mcnamara, 2013). These results suggest that more extensive crosstalk between the peripheral circulatory system and CNS in APOE4 carriers may underlie their long-term differential response to lifestyle factors and contribute to immune and metabolic disruptions during midlife and the onset of AD pathology.

The BBB is important in maintaining the environment of the brain and protecting it from the peripheral circulatory system, which may be compromised in AD. Evidence suggest that APOE4 is associated with BBB leakage via the cyclophilin A (CypA)-matrix metalloproteinase-9 (MMP9) pathway, which leads to the degradation of BBB proteins and loss of pericytes (Montagne et al., 2021). Middle-aged human APOE4 carriers without dementia had higher BBB permeability in the MTL regions than non-carriers, which correlated with cognitive dysfunction and with a CSF vascular marker, soluble platelet-derived growth factor receptor-β (sPDGFRβ), independent of Aβ and tau (Nation et al., 2019). An early-onset AD mice model expressing human APOE4 (E4-FAD) also showed BBB breakdown, reduced cerebral blood flow, neuronal loss and behavioral deficits, compared to E3-FAD mice, and the removal of astrocytic APOE4 in APOE4-KI mice ameliorated leaky BBB and abnormal level of MMP9 transcript (Montagne et al., 2021; Jackson et al., 2022). Moreover, pericyte-deficient mice showed disrupted WM integrity at mid-age following prominent BBB breakdown at early age (Montagne et al., 2018). This may be reflected in our network analysis (Figure 3), where more extensive connectivity between multimodal biomarkers in the APOE4 carriers (i.e., stronger associations between dietary factors or peripheral blood markers and changes in brain WM) suggests that BBB integrity may be compromised.

Our approach to analyzing multimodal variables encompasses both conventional mass univariate correlational analysis and multivariate network analysis, which allowed us to provide a comprehensive overview of the altered peripheral-central interaction. The concept of representing biological systems as networks has been applied to many scientific disciplines (e.g., brain connectivity, metabolic network, gene co-expression analysis) to more efficiently represent complex relationships on the whole system level (Horvath, 2011; Koutrouli et al., 2020). Another strength is the inclusion of longitudinal data from a community-dwelling, ethnic minority Puerto Rican sample with extensive multimodal biological data collected across a 12-year period. Nonetheless, our study is limited to a subsample of BPRHS (the parent study included 1,500 participants in total) with multimodal data available, resulting in relatively small and imbalanced groups, and we were unable to examine the dose-dependent effects of APOE4 (homozygosity vs. heterozygosity) on imaging and cognitive outcomes. The statistical framework we applied sought to mitigate this issue, and generated consistent clusters of baseline fatty acids (indicating robustness of the statistical model) and different connections that are biologically meaningful (e.g., vitamin B12 and omega-3 and brain) (Rathod et al., 2016). Moreover, the BPRHS is currently collecting more (and longitudinal) MRIs, which will bring better insights for explaining potential mechanisms connecting risk factors and the brain.

In conclusion, we showed that APOE4 carrier status is associated with changes in brain WM structure and cognitive performance in both Hispanic and non-Hispanic White older adults without dementia. Moreover, using a combined univariate and network analytical approach, we showed that the association patterns between diet, multimodal blood markers (especially inflammatory cytokines) and brain MTL WM microstructural integrity differ between APOE4 carriers and non-carriers. The comprehensive network model from our study mapped potential key pathways through which APOE4 may interact with diet and immune system and associate with neurobiological changes to increase the risk for AD. Future investigations elaborating on specific biomarkers (e.g., CRP, B vitamins, Klotho, etc.) and pathways (e.g., immune regulation, lipid metabolism) highlighted by our network analysis may promote discovery of novel molecular targets and development of personalized therapeutic strategies for individuals with diverse genetic backgrounds.

## Data availability statement

The datasets presented in this article are not readily available due to ethical restrictions to protect participant privacy. Requests to access the datasets should be directed to the corresponding author.

## Ethics statement

The studies involving humans were approved by University of Massachusetts (UMASS) Lowell Institutional Review Board, IRB #17–143. The studies were conducted in accordance with the local legislation and institutional requirements. De-identified human data was acquired as part of the previously described parent studies for which ethical approval and participant consent were obtained. For the current analysis, written informed consent for participation was not required from the participants or the participants’ legal guardians/next of kin in accordance with the national legislation and institutional requirements.

## Author contributions

YG: Investigation, Methodology, Data curation, Formal analysis, Validation, Visualization, Writing – original draft. CHC: Data curation, Formal analysis, Investigation, Methodology, Software, Visualization, Writing – original draft. LB: Methodology, Software, Writing – review & editing. SN: Data curation, Formal analysis, Methodology, Writing – review & editing. SB: Data curation, Investigation, Writing – review & editing. MG: Data curation, Formal analysis, Investigation, Resources, Writing – review & editing. TS: Data curation, Formal analysis, Funding acquisition, Investigation, Writing – review & editing. JO: Validation, Writing – review & editing. KT: Conceptualization, Funding acquisition, Project administration, Resources, Supervision, Writing – review & editing. RB: Conceptualization, Funding acquisition, Methodology, Resources, Writing – review & editing. B-BK: Conceptualization, Funding acquisition, Investigation, Methodology, Resources, Supervision, Writing – review & editing, Writing – original draft.

## Alzheimer's disease neuroimaging initiative

Part of the data used in preparation of this article were obtained from the Alzheimer's Disease Neuroimaging Initiative (ADNI) database (adni.loni.usc.edu). As such, the investigators within the ADNI contributed to the design and implementation of ADNI and/or provided data but did not participate in analysis or writing of this report. A complete listing of ADNI investigators can be found at: http://adni.loni.usc.edu/wp-content/uploads/how_to_apply/ADNI_Acknowledgement_List.pdf.
