# Corrigendum: APOE4 allele-specific associations between diet, multimodal biomarkers, and cognition among Puerto Rican adults in Massachusetts

**DOI:** 10.3389/fnagi.2023.1343591

**Published:** 2023-12-12

**Authors:** Yi Guan, Chia Hsin Cheng, Luis I. Bellomo, Sriman Narain, Sherman J. Bigornia, Mahdi O. Garelnabi, Tammy Scott, José M. Ordovás, Katherine L. Tucker, Rafeeque Bhadelia, Bang-Bon Koo

**Affiliations:** ^1^Department of Anatomy and Neurobiology, Boston University Chobanian and Avedisian School of Medicine, Boston, MA, United States; ^2^Department of Agriculture, Nutrition, and Food Systems, College of Life Sciences and Agriculture, University of New Hampshire, Durham, NH, United States; ^3^Department of Public Health, Zuckerberg College of Health Sciences, University of Massachusetts Lowell, Lowell, MA, United States; ^4^School of Medicine, Tufts University, Boston, MA, United States; ^5^Nutrition and Genomics Laboratory, J.M.-US Department of Agriculture Human Nutrition Research Center on Aging at Tufts University, Boston, MA, United States; ^6^IMDEA Alimentacion, Madrid, Spain; ^7^CIBER Fisiopatologia de la Obesidad y la Nutricion (CIBEROBN), Instituto de Salud Carlos III, Madrid, Spain; ^8^Department of Biomedical and Nutritional Sciences, Zuckerberg College of Health Sciences, University of Massachusetts Lowell, Lowell, MA, United States; ^9^Center for Population Health, University of Massachusetts Lowell, Lowell, MA, United States; ^10^Neuroradiology Section, Beth Israel Deaconess Medical Center, Harvard Medical School, Boston, MA, United States

**Keywords:** APOE, blood–brain barrier, neurite orientation dispersion and density imaging, diet, neuroinflammation, blood biomarker, aging, cognitive decline

In the published article, there was an error on the scan parameter description for the diffusion MRI.

A correction has been made to section 2.2.2. Diffusion MRI, the first paragraph. This sentence previously stated:

“Diffusion MRI data were obtained from BPRHS participants, including 49 gradient directions for *b*-values 1,000 and 2,000 s/mm^2^ and 60 gradient directions for b-value 3,000 s/mm^2^ (TR = 2,650 ms, TE = 69.2 ms, FA = 90, ST = 2 mm, total slice number = 69, FOV = 24.0, MX = 120 × 120, multiband acceleration factor = 3, arc acceleration factor = 2).”

The corrected sentence appears below:

“Diffusion MRI data were obtained from participants based on the multishell sampling designed to provide shells of radius 1/3, 2/3, and 1 times the max *b*-value, 2,500 s/mm^2^, with 125 directional encodings (TR = 3,400 ms, TE = 73.4 ms, FA = 90, FOV = 24.0, MX = 256 × 256, ST = 2 mm).”

The authors apologize for this error and state that this does not change the scientific conclusions of the article in any way. The original article has been updated.

