# Supplementary material for: APOE4 allele-specific associations between diet, multimodal biomarkers, and cognition among Puerto Rican adults in Massachusetts
Source: Front Aging Neurosci. 2023 Nov 15;15:1285333. doi: 10.3389/fnagi.2023.1285333 (PMC10684694; doi:10.3389/fnagi.2023.1285333)
Supplement: Supplementary file 1 [file Table_1.DOCX]

Supplementary Material

# Supplementary Methods

**Dietary Measure**

Dietary data were collected with a food frequency questionnaire designed for use in this population (Tucker et al., 1998) (Tucker et al., 2010). Diet quality was measured using the Healthy Eating Index (HEI) published by the Dietary Guidelines for Americans 2005 (Guenther et al., 2008). In brief, HEI-2005 is a composite score including components such as intakes of fruits, vegetables, grains, milk, meat and beans, oils, saturated fats, sodium, etc., expressed per 1,000 kilocalories. A higher score from the maximum of 100 suggests higher diet quality. We also calculated an additional score, HEI excluding the oil component, as blood lipid concentrations were also included in other parts of our analysis.

**Blood measures**

Blood samples were collected from participants who completed the interview and analysed as previously described (Tucker et al., 2010). In brief, on the day after interview following a 12-hour fast, blood was collected by a study phlebotomist in an EDTA tube - and transported in a cold container to UMass Lowell for processing. Tumor necrosis factor α (TNFα), interleukin-6 (IL-6), klotho, and fibroblast growth factor 23 (FGF23) were measured by ELISA according to the manufacturer protocols using BioTek’s Epoch Microplate Reader (Gen5 Software empowered). Clinical Chemistry analyzer (EasyRA) was used for the measurement of c-reactive protein (CRP), total cholesterol, LDL cholesterol (LDL), HDL cholesterol (HDL), triglycerides (TG), phosphorus, creatinine, glucose using reagents supplied by the analyzer manufacturer. Vitamin B6, B12, and a total of 26 blood long chain fatty acid levels were measured by gas chromatography (GC) method as previously indicated (Bigornia et al., 2016). The full list of markers can be found in Table 3.

**Clinical measure**

A cardiovascular risk (CVR) score was calculated by counting hypertension and diabetes status. The score ranged from 0 to 2, where a score of 2 was assigned to individuals with both hypertension and diabetes diagnoses, a score of 1 indicated either having hypertension or diabetes, and a score of 0 was assigned to individuals with neither of those conditions. Hypertension was diagnosed based on systolic or diastolic blood pressure (BP) measurements of 140 mmHg or higher, or 90 mmHg or higher, respectively, or by use of antihypertensive medication, on 12-year visit (and on at least one of the three previous visits). Diabetes was diagnosed based on the use of diabetes medication or a fasting blood glucose of 126 mg/dl or higher on 12-year visit (and on at least one of the three previous visits). BMI was calculated for each participant based on height and weight information at the 12-year visit.

**ADNI measure**

Neuroimaging measure: MRI was performed at 1.5-Tesla or 3.0-Tesla using a T1-weighted 3D MP-RAGE sequence.9 The image parameters were TR/TE/TI equal to 2400/3/1000ms, FA = 8, FOV 24 cm, 192×192 in-plane matrix, and ST=1.2 mm. Processing was performed on the downloaded images using FreeSurfer v6.0 (http://surfer.nmr.mgh.harvard.edu/), using procedures with: 1) Motion correction, 2) skull-stripping (or removal of non-brain tissue), 3) intensity normalization, 4) tissue segmentation, and 5) registration to in-built atlas.10 From the above procedures, we extracted volumes of the left and right hippocampus, and total intracranial volume (ICV).

CSF tau measure: As previously described (https://adni.loni.usc.edu/methods/), CSF samples were collected through lumbar puncture and maintained in the -80C freezer by the ADNI Penn Biomarker Core. Total tau (t-Tau) and phosphorylated tau 181 (p-Tau) levels were obtained using the Roche Elecsys cobas e 601 fully automated immunoassay platform. For the current analysis, we selected and downloaded CSF samples acquired matching/near the baseline MRI acquisition time point.

Cognitive measure: MMSE was administered as previously described in the ADNI manual. For the current analysis, we selected and downloaded MMSE acquired matching the baseline MRI acquisition time point.

**Multivariate network analysis**

Studying the interactions between multimodal biomarkers may help to understand the effect of peripheral perturbation (i.e., diet) on the CNS during aging, but statistical analysis of the high dimensional data can be challenging. To understand a complex system underlying cognitive aging in BPRHS, we applied a multi-layer network analysis model to examine the interactions between variables in the different biological domains listed above. The network model was built based on the weighted correlation network analysis concept, which has been widely applied as a data-mining method for studying biological relationships between a large number of variables (i.e., gene co-expression analysis) (Horvath, 2011, Zhang and Horvath, 2005). The network analysis was carried out in the following steps:

- From each of the APOE4 carrier and non-carrier groups, a raw 50-by-50 connectivity matrix, S (undirected and weighted), was obtained through correlation analysis using 6 imaging (WM ND from 6 ROIs) and 44 non-imaging variables (Table 3), where the values of the matrix encoded the connectivity strength between each pair of variables.

$$\boldsymbol{S=}\left[ \boldsymbol{s}_{\boldsymbol{i,j}} \right]$$

where s_i, j_ is the absolute value of partial correlation coefficient with adjusting for age and sex, and ranges from 0 to 1.

- Next, weak/spurious connections were filtered out and stronger connections were emphasized to remove noise and generate more meaningful network architecture. To achieve this, soft thresholding methods based on 1) power function and 2) topological overlap measure (TOM) were applied to the matrix S (Yip and Horvath, 2007). Power function involves raising edge weight by a power of β to emphasize strong correlations and shrink weak correlations, resulting in a matrix, A:

$$\boldsymbol{A=}\left[ \boldsymbol{a}_{\boldsymbol{i,j}} \right]$$

where

$$\boldsymbol{a}_{\boldsymbol{i,j}}\boldsymbol{=}{\boldsymbol{|}\boldsymbol{s}_{\boldsymbol{i,j}}\boldsymbol{|}}^{\boldsymbol{\beta}}$$

and $\beta=6$ for undirected network.

- Then, TOM was applied to the matrix A, which considers neighboring information (how often the i^th^ and j^th^ vertices connect to the same vertices) for graph clustering:

$$\boldsymbol{w}_{\boldsymbol{i,j}}\boldsymbol{=}\frac{\boldsymbol{l}_{\boldsymbol{i,j}}\boldsymbol{+}\boldsymbol{a}_{\boldsymbol{i,j}}}{\boldsymbol{min}\left\{ \boldsymbol{k}_{\boldsymbol{i}}\boldsymbol{,}\boldsymbol{k}_{\boldsymbol{j}} \right\}\boldsymbol{+1-}\boldsymbol{a}_{\boldsymbol{i,j}}}$$

where

$$\boldsymbol{l}_{\boldsymbol{i,j}}\boldsymbol{=}\sum_{u} \boldsymbol{a}_{\boldsymbol{i,u}}\boldsymbol{a}_{\boldsymbol{u,j}}$$

and

$$\boldsymbol{k}_{\boldsymbol{i}}\boldsymbol{=}\sum_{u} \boldsymbol{a}_{\boldsymbol{i,u}}$$

- The resulting 50-by-50 weighted matrix, W, was termed the lower-level network (LLN, Figure 3A&B, dash-lined boxes, lower level), where the number of nodes corresponds to the total number of individual variables and the lines (also called edges) correspond to their connectivity.

$$\boldsymbol{W=}\left[ \boldsymbol{w}_{\boldsymbol{i,j}} \right]$$

- To identify the relationships more effectively between each factor on the whole system level, we applied a dimensional reduction approach to define modules (Q) within the 50 variables. Identifying the biological or symptom clusters within a complex network can be vital for promoting precision analysis in clinical research (Tunis et al., 2003, Alcala-Corona et al., 2021). In brief, variables were categorized into biologically pre-defined domains (i.e., brain WM microstructure, inflammatory cytokines, lipids, etc.) because those from the same domain were strongly correlated with each other. For the 26 baseline fatty acids, because of the large number of variables and their similarity, we further categorized them into clusters using hierarchical cluster analysis. For hierarchical clustering, the total number of clusters was optimized using the Silhouette method (Supplementary Figure S2).
- The connectivity strength (weight of the edge) between each module of variables was defined as the mean weight of all edges connecting the pair of modules and resulting in the upper-level network (ULN), W^ave^ (Figure 3 A&B boxes, upper level).

$$\boldsymbol{W}_{\boldsymbol{q}\boldsymbol{1,q}\boldsymbol{2}}^{\boldsymbol{ave}}\boldsymbol{=mean(}\boldsymbol{W}^{\left( \boldsymbol{q}\boldsymbol{1,q}\boldsymbol{2} \right)}\boldsymbol{)}$$

- To quantitatively compare the connection strength of matching edges between two APOE4 carriers and non-carriers’ networks, we applied a resampling strategy to randomly subsample 34 subjects of each group (equivalent to 80% of the APOE4 carrier group) to perform correlation analysis and construct network, which was repeated for a total of 1,000 iterations. The balanced sampling from each group also allowed us to account for potential bias of the resulted network from imbalanced sample sizes. Due to the skewed distribution of W^ave^, matching connections’ W^ave^ were compared between APOE4 carriers and non-carriers using the Mann-Whitney U test and Mood’s median test. Network analyses were conducted using an in-house pipeline written in R (version 4.2.3).

# Supplementary Figures and Tables

**
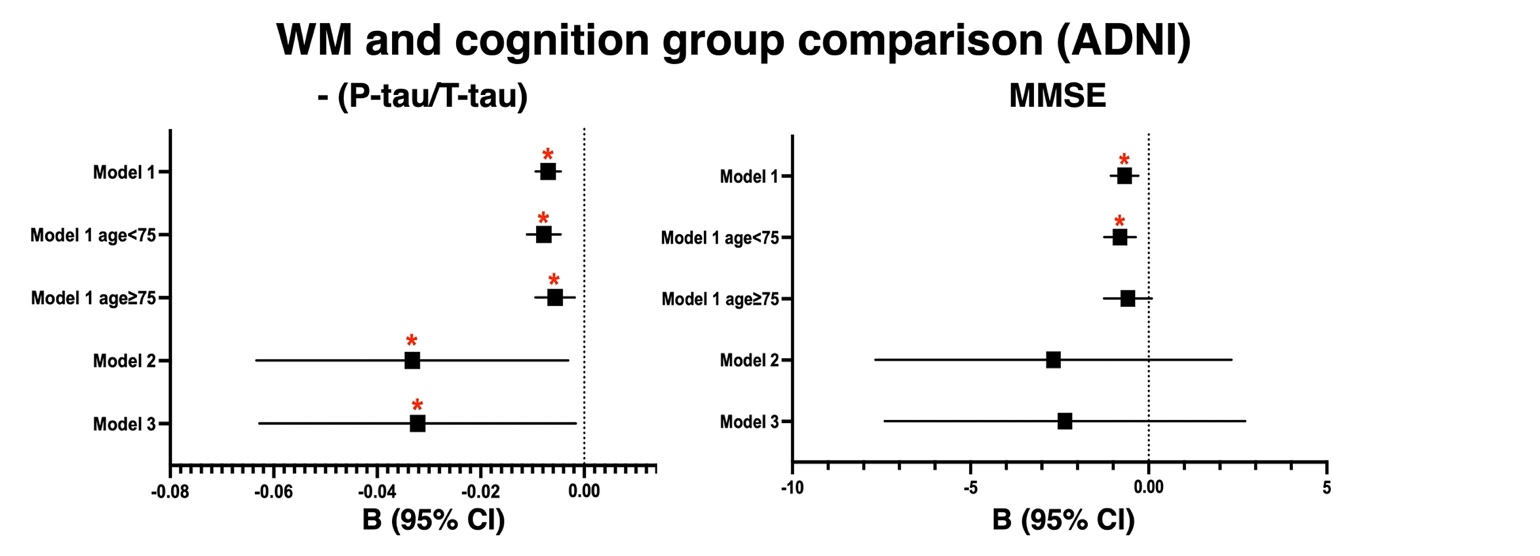
**

**Supplementary Figure S1.** Comparison between APOE4 carriers and non-carriers on WM integrity and MMSE in ADNI. Group-level statistical comparison of APOE4 carriers vs. non-carriers in the WM measure (used CSF P-tau/T-tau as an alternative marker for estimating MTL WM integrity) and MMSE score in the ADNI cohort. We used the inverse value of P-tau/T-tau in this figure to match the direction of MMSE group difference. The linear regression model included APOE4 as the main effect and additional covariates (Model 1: age and sex. Model 2: age, sex, and age×APOE4 interaction. Model 3: age, sex, age×APOE4, education and CVR). Education was included for all models comparing MMSE. *P < 0.05 after correction for multiple comparison using permutation method. Abbreviations: ADNI: Alzheimer’s Disease Neuroimaging Initiative; P-tau: phosphorylated tau; T-tau: total tau; WM = white matter.

**
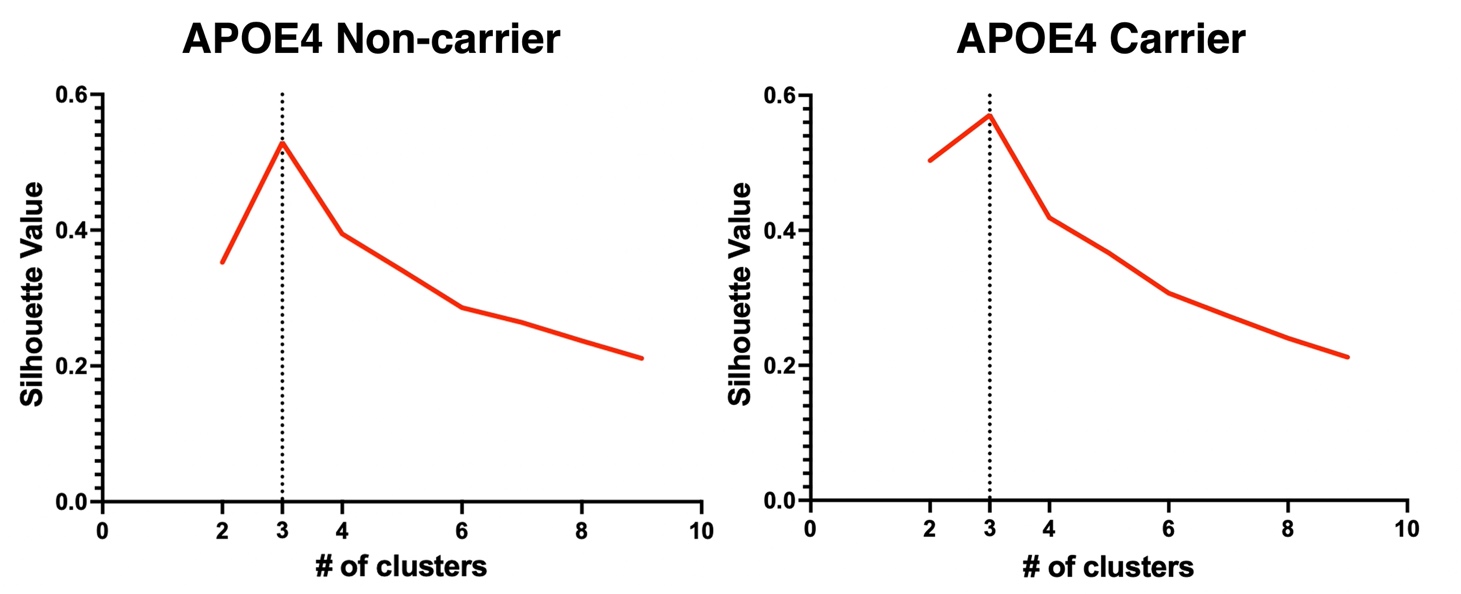
**

**Supplementary Figure S2.** Optimal number of clusters within baseline fatty acids defined by the Silhouette method. Clusters of fatty acids were defined using the hierarchical cluster analysis, and the optimal number of clusters was determined by the peak in the Silhouette curve.

**Table S1. Demographic of ADNI participants**

|  | **ADNI** | | |
| --- | --- | --- | --- |
| **Variable** | APOE4-  (n = 160) | APOE4+  (n = 123) | All  (n = 283) |
| **Age, mean ± SD, y** | 75 ± 5.9 | 74 ± 5.8 | 75 ± 5.9 ^*^ |
| **Hispanic, n (%)** | 0 (0) | 0 (0) | 0 (0) ^*^ |
| **Female, n (%)** | 69 (41) | 44 (36) | 113 (40) ^*^ |
| **Education ≥ 9th grade, n (%)** | 159 (99) | 121 (98) | 280 (99) ^*^ |
| **Hypertension, n (%)** | 67 (42) | 43 (35) | 110 (39) ^*^ |
| **Diabetes, n (%)** | 10 (6.3) | 8 (6.5) | 18 (6.4) ^*^ |
| **BMI, mean ± SD** | 27 ± 4.4 | 26 ± 4.1 | 27 ± 4.3^*^ |

Data are presented as number (percent) or mean ± standard deviation. Abbreviations: APOE4- = non-carriers; APOE4+ = carriers; ADNI = Alzheimer’s Disease Neuroimaging Initiative; BMI = body mass index; ^*^Significantly different compared to BPRHS All (*P* < 0.05) as shown in Table 1.

**Table S2. The association of APOE4 carrier status on brain WM (CSF Tau marker), MMSE, and hippocampal volume in ADNI**

|  | **ADNI (n=283)** | | | | |
| --- | --- | --- | --- | --- | --- |
| **Variable** | **Model 1** | **Model 1** | **Model 1** | **Model 2** | **Model 3** |
| **B (95% CI), P** | **All** | Age < 75 (n=153) | Age ≥ 75 (n=130) |  |  |
| **CSF p-Tau** |  |  |  |  |  |
| p-Tau | **8.4 (5.1, 12)** | **9.1 (4.8, 13)** | **7.8 (2.3, 13)** | 35 (-6.0, 76) | 35 (-6.5, 76) |
|  | **<0**.**0005** | **<0.0005** | **0.006** | 0.094 | 0.098 |
| p-Tau/t-Tau | **0.007 (0.005, 0.009)** | **0.008 (0.005, 0.011)** | **0.006 (0.002, 0.010)** | **0.031 (0.003, 0.063)** | **0.039 (0.002, 0.063)** |
|  | **<0.0005** | **<0.0005** | **0.004** | **0.031** | **0.039** |
| **GM volume** |  |  |  |  |  |
| Left hippocampus | **-276 (-389, -164)** | **-308 (-462, -155)** | **-174 (-324, -26)** | -937 (-2367, 492) | -907 (-2283, 468) |
|  | **<0.0005** | **<0.0005** | **0.02** | 0.20 | 0.20 |
| Right hippocampus | **-321 (-442, -200)** | **-358 (-525, -190)** | **-212 (-364, -59)** | -1085 (-2617, 447) | -994 (-2459, 470) |
|  | **<0.0005** | **<0.0005** | **0.007** | 0.16 | 0.18 |
| Average hippocampus | **-299 (-411, -187)** | **-333 (-587, -179)** | **-193 (-335, -51)** | -1011 (-1433, 411) | -951 (-2307, 405) |
|  | **<0.0005** | **<0.0005** | **0.008** | 0.16 | 0.17 |
| **Cognitive test** |  |  |  |  |  |
| MMSE | **-0.86 (-1.1, -0.29)** | **-0.80 (-1.3, -0.36)** | -0.58 (-1.3, 0.092) | -2.7 (-7.7, 2.3) | -2.4 (-7.4, 2.7) |
|  | **0.001** | **0.0005** | 0.090 | 0.29 | 0.36 |

Statistical test results are presented for APOE4 carriers vs. non-carriers with unstandardized regression coefficient B (95% confidence interval). *P*-values were corrected for multiple comparisons using the permutation-based method. While studying the APOE4 main effect, we adjusted for different set of covariates in 3 models: model 1 (age and sex), model 2 (age, sex, and age×APOE4 interaction), and model 3 (age, sex, age×APOE4, education, and CVR). Additionally, education was included for all models comparing MMSE, and magnet strength and intracranial volume (ICV) were included for analyzing hippocampal volume. Abbreviations: ADNI = Alzheimer’s disease neuroimaging initiative; CSF = cerebrospinal fluid; WM = white matter; GM = grey matter; MMSE = mini-mental state examination.

**Table S3. List of significant correlations between multimodal variables in APOE4 non-carriers**

|  | **Variable 1** | **Variable 2** | **R** | **P-value** |
| --- | --- | --- | --- | --- |
| **Baseline to 12-year WM** | Arachidic acid | WM #3 | -0.23 | 0.019 |
|  | Oleic acid | WM #6 | -0.21 | 0.028 |
|  | DPAn3 | WM #3 | -0.21 | 0.031 |
|  | Trans oleic acid | WM #5 | 0.19 | 0.044 |
| **Baseline to 12-year clinical/blood** | Linoleic acid | HDL | 0.32 | 0.001 |
|  | Total n6 | HDL | 0.32 | 0.001 |
|  | Nervonic acid | Glucose | -0.30 | 0.002 |
|  | DPAn6 | Fgf23 | 0.32 | 0.002 |
|  | Linoleic acid | CVR | -0.27 | 0.003 |
|  | Total n6 | TG | -0.28 | 0.005 |
|  | B6 | Fgf23 | -0.17 | 0.008 |
|  | Dihomo-GLA | CVR | -0.25 | 0.008 |
|  | Stearic acid | Klotho | -0.26 | 0.009 |
|  | DHA | HDL | -0.26 | 0.009 |
|  | Arachidonic acid | LDL | -0.25 | 0.010 |
|  | Total n3 | Fgf23 | -0.25 | 0.010 |
|  | Eicosadienoic acid | TG | -0.25 | 0.011 |
|  | Arachidonic acid | Cholesterol | -0.26 | 0.011 |
|  | DPAn3 | CVR | 0.25 | 0.011 |
|  | Behenic acid | Klotho | 0.27 | 0.012 |
|  | EPA | Fgf23 | -0.20 | 0.018 |
|  | Oleic acid | Phosphorus | 0.24 | 0.019 |
|  | Eicosadienoic acid | HDL | 0.23 | 0.019 |
|  | Behenic acid | Glucose | -0.22 | 0.019 |
|  | Behenic acid | TG | -0.21 | 0.022 |
|  | Docosatetraenoic acid | Fgf23 | 0.23 | 0.022 |
|  | B6 | BMI | -0.21 | 0.023 |
|  | Lignoceric acid | Glucose | -0.23 | 0.024 |
|  | Arachidic acid | Glucose | 0.22 | 0.028 |
|  | Stearic acid | Glucose | 0.22 | 0.029 |
|  | DPAn6 | HDL | -0.21 | 0.030 |
|  | Palmitic acid | CRP | -0.21 | 0.032 |
|  | Linoleic acid | CRP | -0.21 | 0.032 |
|  | Nervonic acid | TG | -0.20 | 0.032 |
|  | Trans palmitoleic acid | CRP | -0.20 | 0.033 |
|  | Stearic acid | CRP | 0.21 | 0.035 |
|  | Linoleic acid | Cholesterol | 0.21 | 0.035 |
|  | Trans palmitoleic acid | TG | -0.20 | 0.036 |
|  | DHA | Fgf23 | -0.21 | 0.036 |
|  | Linoleic acid | LDL | 0.21 | 0.040 |
|  | HEI1 | IL-6 | -0.21 | 0.041 |
|  | Lignoceric acid | HDL | -0.20 | 0.041 |
|  | Dihomo-GLA | Klotho | 0.21 | 0.044 |
|  | Total n3 | HDL | -0.20 | 0.045 |
|  | Stearic acid | Creatinine | 0.19 | 0.048 |
|  | Arachidonic acid | CRP | 0.20 | 0.048 |
|  | Total n6 | CVR | -0.19 | 0.049 |
|  | CVR | WM #1 | -0.33 | <0.0005 |
|  | CVR | WM #3 | -0.35 | <0.0005 |
|  | CVR | WM #4 | -0.34 | <0.0005 |
|  | CVR | WM #2 | -0.32 | 0.001 |
|  | CVR | WM #5 | -0.26 | 0.005 |
| 12-year clinical/blood to 12-year WM | Creatinine | WM #2 | -0.28 | 0.006 |
|  | Creatinine | WM #4 | -0.26 | 0.008 |
|  | Creatinine | WM #3 | -0.24 | 0.012 |
|  | Creatinine | WM #6 | -0.25 | 0.013 |
|  | Creatinine | WM #1 | -0.23 | 0.017 |
|  | Klotho | WM #6 | 0.20 | 0.036 |
|  | CVR | WM #6 | -0.20 | 0.037 |

Statistical test results are presented for APOE4 non-carriers with correlation (adjusted for age and sex) coefficient R and *P*-value corrected for multiple comparisons using permutation method. Due to the large number of tests performed, only significant correlations were presented in this table and were ranked within each category based on the level of significance using *P*<0.05. Abbreviations: WM = white matter; WM #1/2 = left/right inferior fronto-occipital fasciculus; WM #3/4 = left/right inferior longitudinal fasciculus; WM #5/6 = left/right superior longitudinal fasciculus temporal portion. A full list of abbreviations can be found in Table 3 and S5.

**Table S4. List of significant correlations between multimodal variables in APOE4 carriers**

| **APOE4 carriers** | **Variable 1** | **Variable 2** | **R** | **P-value** |
| --- | --- | --- | --- | --- |
| **Baseline to 12-year WM** | B12 | WM #4 | 0.38 | 0.010 |
|  | GLA | WM #1 | -0.40 | 0.013 |
|  | B6 | WM #3 | 0.34 | 0.014 |
|  | GLA | WM #3 | -0.37 | 0.022 |
|  | Total n3 | WM #4 | 0.35 | 0.025 |
|  | Docosatetraenoic acid | WM #6 | -0.35 | 0.026 |
|  | DHA | WM #4 | 0.34 | 0.026 |
|  | Docosatetraenoic acid | WM #5 | -0.36 | 0.028 |
|  | HEI1 | WM #5 | 0.32 | 0.039 |
|  | Docosatetraenoic acid | WM #3 | -0.33 | 0.042 |
| **Baseline to 12-year clinical/blood** | Stearic acid | Phosphorus | -0.65 | <0.0005 |
|  | DPAn3 | Phosphorus | -0.44 | 0.007 |
|  | Palmitoleic acid | Phosphorus | 0.43 | 0.010 |
|  | Palmitic acid | Fgf23 | -0.41 | 0.011 |
|  | DPAn6 | Cholesterol | -0.41 | 0.013 |
|  | Linoleic acid | Fgf23 | -0.40 | 0.019 |
|  | Myristic acid | HDL | 0.42 | 0.025 |
|  | HEI | Fgf23 | -0.34 | 0.030 |
|  | Total n6 | Phosphorus | 0.33 | 0.032 |
|  | HEI1 | Fgf23 | -0.33 | 0.034 |
|  | B12 | CRP | 0.35 | 0.034 |
|  | Trans linoleic acid | IL-6 | -0.31 | 0.037 |
|  | GLA | CVR | 0.35 | 0.037 |
|  | Myristic acid | Fgf23 | 0.21 | 0.038 |
|  | DPAn6 | HDL | -0.34 | 0.040 |
|  | A-Linolenic acid | IL-6 | -0.31 | 0.044 |
|  | DHA | Cholesterol | 0.34 | 0.044 |
|  | Docosatetraenoic acid | Cholesterol | -0.33 | 0.047 |
|  | DPAn6 | Fgf23 | 0.33 | 0.047 |
| **12-year clinical/blood to 12-year WM** | CRP | WM #6 | -0.50 | 0.001 |
|  | Creatinine | WM #3 | -0.49 | 0.002 |
|  | CRP | WM #5 | -0.46 | 0.006 |
|  | Creatinine | WM #1 | -0.42 | 0.007 |
|  | TG | WM #1 | -0.39 | 0.017 |
|  | CRP | WM #1 | -0.38 | 0.019 |
|  | TG | WM #3 | -0.39 | 0.020 |
|  | TNFa | WM #3 | -0.36 | 0.024 |
|  | TG | WM #2 | -0.35 | 0.040 |
|  | HDL | WM #3 | 0.32 | 0.040 |
|  | CVR | WM #3 | -0.33 | 0.041 |
|  | CRP | WM #2 | -0.34 | 0.049 |
|  | CRP | WM #3 | -0.32 | 0.049 |

Statistical test results are presented for APOE4 carriers with correlation (adjusted for age and sex) coefficient R and P-value corrected for multiple comparisons using permutation method. Due to the large number of tests performed, only significant correlations were presented in this table and were ranked within each category based on the level of significance using *P*<0.05. Abbreviations: WM = white matter; WM #1/2 = left/right inferior fronto-occipital fasciculus; WM #3/4 = left/right inferior longitudinal fasciculus; WM #5/6 = left/right superior longitudinal fasciculus temporal portion. A full list of abbreviations can be found in Table 3 and S5.

**Table S5. Baseline blood fatty acid measures**

| **Number Code** | **Fatty Acid Name** | **APOE4- (n=113)** | | **APOE4+ (n=43)** | | **B**  **(95% CI)** |
| --- | --- | --- | --- | --- | --- | --- |
|  |  | Median (IQR) | Cluster | Median (IQR) | Cluster |  |
| 1 | Myristic C14:0 | 0.26 (0.20-0.32) | 1 | 0.28 (0.23-0.35) | 1 | 0.0436  (-0.005, 0.077) |
| 2 | Palmitic C16:0 | 22 (21-22) | 1 | 22 (21-22) | 1 | 0.26  (-0.17, 0.68) |
| 3 | Trans Palmitoleic C16:1n7t | 0.13 (0.11-0.15) | 1 | 0.12 (0.11-0.16) | 1 | 0.003  (-0.011, 0.016) |
| 4 | Trans Oleic C18:1t | 0.99 (0.85-1.2) | 1 | 1.0 (0.83-1.3) | 1 | 0.044  (-0.079, 0.17) |
| 5 | Trans Linoleic C18:2n6t | 0.16 (0.14-0.19) | 1 | 0.17 (0.14-0.20) | 1 | 0.014  (-0.0003, 0.029) |
| 6 | Palmitoleic C16:1n7 | 0.45 (0.34-0.63) | 1 | 0.45 (0.33-0.66) | 1 | 0.015  (-0.066, 0.097) |
| 7 | Stearic C18:0 | 17 (17-18) | 1 | 18 (17-18) | 1 | 0.048  (-0.29, 0.38) |
| 8 | Oleic C18:1n9 | 14 (14-15) | 1 | 15 (14-15) | 1 | 0.076  (-0.41, 0.56) |
| 9 | Linoleic C18:2n6 | 12 (11-13) | 1 | 12 (11-13) | 1 | 0.20  (-0.39, 0.80) |
| 10 | G-Linolenic (GLA) C18:3n6 | 0.14 (0.12-0.17) | 1 | 0.15 (0.13-0.17) | 1 | 0.002  (-0.012, 0.017) |
| 11 | Arachidic C20:0 | 0.19 (0.17-0.21) | 1 | 0.18 (0.16-0.21) | 1 | **-0.015***  **(-0.030, -0.001)** |
| 12 | A-Linolenic C18:3n3 | 0.12 (0.10-0.16) | 1 | 0.14 (0.10-0.17) | 1 | 0.013  (-0.005, 0.031) |
| 13 | Eicosenoic C20:1n9 | 0.21 (0.19-0.24) | 1 | 0.22 (0.19-0.23) | 1 | -0.004  (-0.016, 0.008) |
| 14 | Eicosadienoic C20:2n6 | 0.31 (0.29-0.34) | 1 | 0.30 (0.27-0.33) | 1 | -0.009  (-0.026, 0.008) |
| 15 | Dihomo-GLA C20:3n6 | 1.8 (1.6-2.0) | 1 | 1.8 (1.6-2.0) | 1 | -0.013  (-0.14, 0.11) |
| 16 | Behenic C22:0 | 0.24 (0.21-0.29) | 2 | 0.23 (0.20-0.30) | 2 | 0.0002  (-0.027, 0.027) |
| 17 | Arachidonic C20:4n6 | 17 (16-18) | 1 | 17 (15-18) | 1 | 0.357  (-0.884, 0.171) |
| 18 | EPA C20:5n3 | 0.39 (0.30-0.49) | 1 | 0.38 (0.32-0.47) | 1 | -0.017  (-0.091, 0.057) |
| 19 | Lignoceric C24:0 | 0.55 (0.45-0.66) | 2 | 0.56 (0.43-0.69) | 2 | 0.0004  (-0.058, 0.059) |
| 20 | Docosatetraenoic C22:4n6 | 3.7 (3.3-4.1) | 1 | 4.0 (3.3-4.2) | 1 | -0.027  (-0.26, 0.21) |
| 21 | Nervonic C24:1n9 | 0.53 (0.44-0.61) | 2 | 0.51 (0.38-0.61) | 2 | -0.016  (-0.074, 0.043) |
| 22 | Docosapentaenoic C22:5n6 (DPAn6) | 0.78 (0.69-0.89) | 1 | 0.80 (0.71-0.94) | 1 | 0.011  (-0.049, 0.071) |
| 23 | Docosapentaenoic C22:5n3 (DPAn3) | 2.0 (1.8-2.2) | 1 | 1.9 (1.8-2.1) | 1 | -0.041  (-0.16, 0.080) |
| 24 | DHA C22:6n3 | 4.1 (3.5-4.7) | 3 | 4.0 (3.3-4.4) | 3 | -0.23  (-0.59, 0.13) |
| 25 | Total n3 (EPA, DPA, DHA) | 6.5 (5.9-7.0) | 3 | 6.2 (5.4-6.9) | 3 | -0.29  (-0.72, 0.15) |
| 26 | Total n6 | 31 (30-32) | 1 | 32 (30-32) | 1 | -0.17  (-0.73, 0.39) |

The list of all 26 fatty acids from the blood analysis with their corresponding number code used in the network analysis in Figure 3. Data for each group is presented as median (interquartile range). The cluster numbers were assigned to each fatty acid using the hierarchical cluster analysis and the optimal number of clusters was determined using the Silhouette method. Statistical group comparison between APOE4 carriers and non-carriers were conducted with linear regression model adjusting for age and sex. *Significantly different between APOE4 carriers vs. non-carriers with *P*<0.05.

**References**

ALCALA-CORONA, S. A., SANDOVAL-MOTTA, S., ESPINAL-ENRIQUEZ, J. & HERNANDEZ-LEMUS, E. 2021. Modularity in Biological Networks. *Front Genet,* 12**,** 701331.

BIGORNIA, S. J., HARRIS, W. S., FALCON, L. M., ORDOVAS, J. M., LAI, C. Q. & TUCKER, K. L. 2016. The Omega-3 Index Is Inversely Associated with Depressive Symptoms among Individuals with Elevated Oxidative Stress Biomarkers. *J Nutr,* 146**,** 758-66.

GUENTHER, P. M., REEDY, J. & KREBS-SMITH, S. M. 2008. Development of the Healthy Eating Index-2005. *J Am Diet Assoc,* 108**,** 1896-901.

HORVATH, S. 2011. *Weighted network analysis: applications in genomics and systems biology.*, Springer Science & Business Media.

TUCKER, K. L., BIANCHI, L. A., MARAS, J. & BERMUDEZ, O. I. 1998. Adaptation of a food frequency questionnaire to assess diets of Puerto Rican and non-Hispanic adults. *Am J Epidemiol,* 148**,** 507-18.

TUCKER, K. L., MATTEI, J., NOEL, S. E., COLLADO, B. M., MENDEZ, J., NELSON, J., GRIFFITH, J., ORDOVAS, J. M. & FALCON, L. M. 2010. The Boston Puerto Rican Health Study, a longitudinal cohort study on health disparities in Puerto Rican adults: challenges and opportunities. *BMC Public Health,* 10**,** 107.

TUNIS, S. R., STRYER, D. B. & CLANCY, C. M. 2003. Practical clinical trials: increasing the value of clinical research for decision making in clinical and health policy. *JAMA,* 290**,** 1624-32.

YIP, A. M. & HORVATH, S. 2007. Gene network interconnectedness and the generalized topological overlap measure. *BMC Bioinformatics,* 8**,** 22.

ZHANG, B. & HORVATH, S. 2005. A general framework for weighted gene co-expression network analysis. *Stat Appl Genet Mol Biol,* 4**,** Article17.
